# Sample size justification in feasibility studies: moving beyond published guidance

**DOI:** 10.1186/s40814-025-01675-9

**Published:** 2025-06-23

**Authors:** Robert Montgomery

**Affiliations:** Department of Biostatistics and Data Science, University of Kansas Medical Center, 3901 Rainbow Blvd., Kansas City, 66160 KS USA

**Keywords:** Sample size, Pilot study, Feasibility, Rules of thumb, Guidance

## Abstract

Pilot and feasibility studies are crucial for determining whether follow-up trials should be conducted. To be effective, these studies need to be properly designed and have large enough samples to make correct decisions about proceeding to a future trial at a high rate. However, there is significantly less clarity about best practices concerning sample size justifications compared to larger randomized controlled trials which are usually justified by power analyses. This has led to many feasibility studies justifying sample sizes based on pragmatic reasons (e.g., the ability to complete) or on published guidance and various rules of thumb. We argue that feasibility studies should be designed for, or at least report relevant operating characteristics, specifically, the probability of determining a future trial will be feasible when it is and the probability of determining a trial will be feasible when it is not and that sample size justifications should be based on these operating characteristics whenever possible. If relevant operating characteristics for a study are not reported, it is difficult to know whether the evidence from these trials can be used to claim a future trial would be feasible. In this paper, we review common methods for determining samples sizes in feasibility studies, report a narrative review of recently published feasibility studies and use two case-studies to highlight core issues we believe exist in the current design and reporting of these studies. Finally, we provide some suggestions that we believe will enhance the sample size justifications and overall effectiveness of these important trials.

**Supplementary information:**

The online version contains supplementary material available at 10.1186/s40814-025-01675-9.

## Introduction

Randomized controlled trials (RCTs) are considered the gold standard for evaluating the effectiveness of an intervention. Before conducting these larger confirmatory trials research teams often conduct a series of smaller trials to evaluate whether the intervention is safe, has the potential to be effective, and whether it is feasible to conduct the RCT. The design, analysis plan, and sample size of these early-stage studies needs to be sufficiently justified to effectively discriminate between feasible and infeasible RCTs. However, the sample size justification for feasibility studies has traditionally been less rigorous, and therefore less clear, compared to the justification of sample sizes in RCTs. Sample sizes for RCTs are often justified by the frequentist operating characteristics (OCs) they provide (typically type I error rate and power), and it is routine to see statements such as “The sample size was chosen to achieve 80% power $\dots$”. This provides some assurance that if (sometimes a big if) the assumptions underlying the sample size justification are correct then the study will come to the correct conclusion most of the time. This differs greatly from how sample sizes are chosen in feasibility studies where the sample size is often chosen based on previous studies, published recommendations, or pragmatic reasons. There are obvious differences between feasibility studies and RCTs, including the objectives, available resources and acceptable error rates; nevertheless, the sample sizes for feasibility studies should also be sufficient to address their objectives at an acceptable rate and therefore, the justification of the sample sizes should provide some assurance that there is a high probability of making the correct determination of whether a future trial is feasible.

Feasibility studies are complex trials with typical objectives of estimating values to power a future RCT, determining if an intervention is acceptable to participants and assessing whether a future RCT would be feasible based on measures such as retention and recruitment rate. These objectives are often interconnected, for example acceptability to participants is likely associated with retention which is directly associated with the required sample size for a future RCT. Feasibility studies are expected to evaluate these complex outcomes with typically small sample sizes. However, or perhaps because of this complexity, study teams seem to default to providing no reasoning for sample sizes, or outsourcing the reasoning to guidelines and recommendations that may not be relevant for the study at hand. This approach among researchers toward sample sizes for feasibility studies is at odds with other developments regarding these early-stage studies which have seen a dramatic increase in attention. From 2013 to 2023, the results for the terms ‘‘pilot’’ and ‘‘feasibility’’ doubled in a PubMed search. Additionally, in 2016, the Consolidated Standard of Reporting Trials (CONSORT) extension to pilot and feasibility studies was published [1] and provided further guidance to researchers by clarifying that the primary aim of these studies “is to assess [the] feasibility of conducting the future definitive RCT”. In the same year Eldridge et al. [2], noting the heterogeneity in these studies, published a conceptual framework that clarified the similarities and differences between studies labeled as ‘‘pilot’’ or ‘‘feasibility’’, with the conclusion that pilots are a subset of feasibility studies that in some way mimic a future trial. More recently this framework was extended to include internal pilot studies and discuss the levels of uncertainty that may influence the outcomes of these trials. [3]. There has also been recent interest in developing methodological tools to assess feasibility outcomes, with both frequentist and Bayesian methods proposed that allow researchers to move beyond simple comparisons to a threshold. Considering these other efforts to improve feasibility studies, the relative lack of progress on determining and justifying sample sizes is striking.

In this article, we review common methods for determining sample sizes, including rules of thumb, and discuss current sample sizes in the literature and how they are justified. Given that formal analysis of feasibility outcomes is not widespread we also review recently proposed methods for assessing these outcomes. We use two recently published feasibility protocols as case-studies to highlight common limitations and offer suggestions to improve the overall quality of sample size determination in feasibility studies.

## Sample sizes in pilot and feasibility studies

### Guidance and rules of thumb

Many authors have provided minimum sample size guidance and rules of thumb for external pilot and feasibility studies based on estimating the standard deviation to power a future trial, including Julious [4] who suggested 12 per arm, Teare et al. [5] who suggested 35 and 60 per arm depending on whether the outcomes were continuous or binary and Sim and Lewis [6] who suggested a sample of “at least *n* = 50”. Sample size approaches not based on the standard deviation still typically focus on a single parameter of interest. For example, Viechtbauer et al. [7] provided guidance on choosing a sample size to achieve a probability of detecting a problem on a single outcome.

These sample size suggestions are correct, in the sense that they will perform well if their assumptions hold, and the objective of a feasibility study is only to estimate the specified parameter of interest for which the guidance was derived. Given the complexity of feasibility studies, with many outcomes of interest, and the increased focus on feasibility as the primary outcome during the time since many of these recommendations were published it is unlikely they are “correct” for many of the studies that use them.

Even when a trial collects an outcome that is covered by published guidance, the guidance may not be an appropriate basis for the sample size of the entire trial. For example, consider a two-arm feasibility study collecting data on the recruitment rate and estimating the standard deviation. Based on guidance from Julious [4] *N* = 24 may be sufficient to estimate the standard deviation. Further assume that the target recruitment rate is 20 participants per month, then the observed recruitment rate can be used to assess the feasibility of recruitment. However, a simulation (details provided in the Supplementary information) shows that with *N* = 24 the estimation of this monthly recruitment rate would be highly variable, specifically there would be a 21% chance that the estimated recruitment rate would differ from the true rate of 20 per month by 5 or more (i.e., 20% of the estimated recruitment rates will be $\le 15$ or $\ge 25$ per month) which would have obvious implications for a real study. If we increase the sample size in the simulation to *N* = 50 the probability of the estimate being off by 5 or more would drop to only 9%. For this example, the rule of thumb provided by Julious is applicable for part of the study, but it may not provide satisfactory performance for all objectives of the trial. Unless other justification is provided it is not clear that guidance or rules of thumb will provide sufficient sample sizes for feasibility studies with multiple outcomes of interest.

### Current state of sample size justifications

Totton et al. [8] conducted a systematic review of randomized controlled pilot and feasibility studies in the United Kingdom from 2013 to 2020 and identified 761 studies with a median sample size of 60 (IQR 40–100) across two arms. They then compared the observed sample sizes to commonly used rules of thumb for estimating standard deviations and found that over half would meet the recommendations of $\ge 55$ across the entire study suggested by Sim and Lewis and that 99% met the least stringent suggestion for 10 participants per arm [9]. The authors noted that most studies did not meet more recent recommendations and that the majority of studies failed to fully recruit. They concluded, regarding rules of thumb for estimating standard deviations, that “the information collected may not be sufficient.” Unfortunately, they did not report the number of feasibility outcomes collected or how they sample sizes were determined which makes it unclear what proportion of the use of rules of thumb was appropriate. In a literature survey of 26 pilot and feasibility studies from 2007 to 2008 Arain et al. [10] found that only 35% had a sample size calculation; they also found that 81% of studies used hypothesis tests for efficacy outcomes making it unclear how many, if any, sample sizes were determined by feasibility. We conducted a brief survey of the first 20 published protocols or results articles (excluding solely qualitative studies, reviews, and methodological papers) published in the journal Pilot and Feasibility Studies in 2024. Table 1 provides descriptive statistics of these studies and details of the studies are available in Supplementary Table S1. This survey is not intended to be systematic, and given it comes from one journal may not be representative of all feasibility studies. The goal of the survey was to show that it is common for feasibility studies to be justified without regard to all outcomes. Of the twenty papers, 19 (95%) either stated, or it was clear from the text that feasibility was one of the primary objectives. The studies reported a median of four quantitatively assessed feasibility outcomes (e.g., proportions, rates), with an inter quartile range of (3.0-5.25). If one, or several outcomes were labeled “primary” or somehow designated as being progression criteria (e.g., in a table labeled “Key feasibility indicators for progression”), only those variables were counted. Additionally, qualitative feasibility outcomes were not included resulting in a conservative estimate of the number of feasibility outcomes assessed in these studies. 

Traditional rules of thumb were the most common sample size justification (8/25), followed by studies with unclear reasoning, or none given (5/25). Two studies determined the sample size based on hypothesis tests for efficacy outcomes and the three other studies were based on (1) the percentage of a full trial for an internal pilot, (2) previous papers having used similar sample sizes, and (3) pragmatic considerations. Only two studies defined the sample size based on assessing feasibility even though 95% of the studies listed feasibility as a primary objective. These two studies used the precision of the confidence interval around a feasibility outcome and hypothesis tests for feasibility outcomes respectively.

The use of rules of thumb was heterogeneous between studies, some included a single rule of thumb while others cited guidance from several different articles to show their sample was within a range of common sample sizes. One concerning aspect of the use of rules of thumb in general is that nuance is often lost as the cited articles become standard justifications for specific values. In this survey the most cited guidance was from Brown [11] as justification for *N* = 30. However, reading Browne’s paper we find that the use of this paper as blanket justification for *N* = 30 may not be appropriate. When discussing power loss in a future RCT Browne states ‘‘the proverbial rule of thumb of ‘‘use an *n *of 30 or greater to estimate a parameter’’ will not alleviate that problem, unless the effect size is also quite large” [11]. Despite this nuanced statement, *N* = 30 may not be enough unless the effect size is at least 0.75 (based on results in the original paper), this paper has become a common justification for *N* = 30. This confusion in the literature potentially stems from Lancaster et al.’s comment in their widely cited recommendations for good practice that “A general rule of thumb is to take 30 patients or greater to estimate a parameter (Browne 1995)’’ [12]. This is an easy oversimplification to make and no author, or reviewer, can be expected to have a comprehensive knowledge of all guidance and methods proposed to evaluate feasibility studies. For this reason alone, a simple citation to published guidance as justification for a sample size should be viewed skeptically, a study team needs to argue why that guidance is appropriate and sufficient for their study.

**Table 1:** Results from twenty recent pilot and feasibility studies

	*N* (%)	
**Feasibility as primary outcome**	19 (95)	
**Number of outcomes**	4, IQR: (3.0–5.25)	
**Justification for sample size**		
Rule of thumb	8 (40)	
None given	3 (15)	
Unclear	2 (10)	
Based on feasibility outcomes	2 (10)	
Power for hypothesis test	2 (10)	
Percent of RCT	1 (5)	
Previous studies	1 (5)	
Pragmatic considerations	1 (5)	

## Methods for analyzing feasibility outcomes

If feasibility is the primary aim of a feasibility study as suggested by the CONSORT extension, then the sample size should be based, at least in part, on analyzing feasibility outcomes and they way these outcomes are analyzed will drive the sample size. Approaches for analyzing feasibility outcomes can be broadly categorized into methods based on a threshold, frequentist hypothesis tests, and Bayesian methods.

A common way of assessing feasibility is to compare an observed point estimate, for example, a proportion, to a pre-specified threshold. If the observed value meets or exceeds the thresholds (e.g., retention rate $\ge$ 0.80), then a decision to proceed would be made; otherwise, a future trial would not be pursued. This method is commonly stated in the literature but has poor performance. For example, if the true retention rate in the population is 0.80, meaning the future trial is feasible, then the sample proportion will only meet or exceed the target threshold 50% of the time, and this issue is exacerbated by multiple outcomes, see [13] for more details and simulation study showing the poor performance of the threshold approach.

Avery et al. [14] suggested a related approach that splits the domain of the point estimate into three zones. If the point estimate is in the Red zone, a future trial will not be pursued, in the Amber zone, an RCT might be pursued if adequate trial modifications can be made and in the Green zone, a future trial will be pursued. With retention, the zones might be delineated as Red ($$< 0.70$$), Amber $$[0.70 - 0.80)$$ and Green ($$\ge$$ 0.80). This approach is often referred to as a traffic light procedure. For the traffic light procedure, the decision in the Amber zone is subjective (it rests on whether an underperforming aspect of the trial can be appropriately modified). Lewis et al. [15] proposed an approach where the decision is made as before in the Red or Green zone, but in the Amber zone a decision to proceed is made only if the p-value for a binomial test is less than $$\alpha$$ (the null hypothesis for this test is that the true parameter equals the Red threshold). The authors suggested that for multiple outcomes the type I error rate of the hypothesis test in the Amber zone should be unadjusted, but power for each outcome should be increased to maintain acceptable overall power. For example, if 80% joint power (the probability of determining you would proceed on all outcomes) was set for four feasibility outcomes then power of $$\root 4 \of {0.80} = 0.95$$ would be required for each outcome. However, Montgomery et al. suggested the $$\alpha$$ level should also be adjusted to be $$\root m \of {\alpha }$$ where *m* is the number of outcomes which results in less conservative performance with multiple outcomes. The procedure is applicable to binomial outcomes that the study teams views as modifiable and Lewis et al. provided required sample sizes for a wide range for Red and Green zone limits.

Wilson et al. [16] proposed a test for jointly assessing recruitment, follow-up, and adherence rates. The test uses the relationships between the three parameters relative to the prespecified power of a definitive RCT. A critical value is used as a decision rule to proceed based on the power of a future trial, for example if the estimated power of a future RCT was 80% a decision could be made to proceed, while if it were only 65% a larger trial would not be pursued. The test can only be used for a trial that collects a subset of recruitment, follow-up, and adherence rates and makes a strong assumption about the relationship between adherence and effectiveness. Nevertheless, the authors showed that their test had good power and type I error rate control for most scenarios with approximately 50 subjects per arm and is a compelling option if the feasibility outcomes are a subset of recruitment, adherence, and follow-up.

Wilson et al. [17] also proposed a Bayesian method for assessing the traffic light procedure by minimizing the expected loss of making an error (e.g., proceeding, a Green zone decision, when the true parameter is in the Amber or Red zone). Their approach allows for tradeoffs between the types of errors that can be made but is computationally expensive and requires input from researchers for prior elicitation and loss function determination which may limit its widespread use.

Recently, Montgomery et al. [13] proposed a Bayesian-Frequentist hybrid approach that requires the specification of a set of parameters that would render a future trial feasible denoted by *F* and a set of parameters that would render a future trial infeasible denoted by *I*. They showed their approach was more efficient than other common methods such as Lewis et al.’s hypothesis test procedure and can incorporate a more diverse range of outcomes including proportions, rates, and continuous variables. The procedure provides a probability of proceeding conditional on the feasible and infeasible set of parameter values denoted by $P(P\mid F)$ and $P(P \mid I)$ respectively which are interpreted as the probability of proceeding when a future RCT is feasible and when it is not.

These approaches have various strengths and weaknesses, but their performance will increase as the sample size increases. Therefore, if feasibility is the primary outcome of a pilot or feasibility study, and feasibility can be formally assessed by one or more methods, then it seems to follow that the sample size should be justified based at least in part on the operating characteristics the analysis method provides. Even in situations where the sample size is fixed due to the budget or logistical constraints the operating characteristics (e.g., probability of proceeding when a future RCT is feasible) at the fixed sample size can still be presented for greater transparency. If the sample sizes for feasibility studies are not based on the operating characteristics they provide then there is no guarantee the study will be able to effectively evaluate the outcomes. We note here that this is not so different a recommendation from the published guidance discussed in the “Guidance and rules of thumb” section. For instance, Browne focused on sample sizes required to achieve “80 per cent chance of achieving the planned power in the clinical trial” [11], this is an OC, a desired 80% probability of having 80% power in a future trial. Here, we simply suggest that the OCs of all feasibility outcomes should also be assessed and in general the maximum sample size required for the objectives of a feasibility study should be used.

## Case-study of two recent feasibility protocols

Here we highlight two issues related to the sample size of a feasibility study that are often overlooked.

### Progression criteria need to be meaningful

Davis et al. [18] recently published the protocol for a randomized controlled trial of an intervention to support workers returning from a long-term absence. The proposed study aims to recruit *N* = 100 subjects, based the Medical Research Council’s 2008 guidance for complex interventions [19]. The study will collect many outcomes, but the authors clearly state, “The primary outcome of this pilot trial is to determine its feasibility for a main trial.” The decision will be based on a traffic light system with four progression which are listed in Table 2.

**Table 2:** Progression criteria for Davis et al

Outcome	Green limit	Red limit
Recruitment (proportion screened)	$\ge 0.50$	$< 0.25$
Retention	$\ge 0.70$	$< 0.40$
Intervention Completion	$\ge 0.50$	$<0.25$
Usability of materials	$\ge 0.50$	$<0.25$

The authors should be commended for a clear description of the go/no-go criteria for their key feasibility outcomes, stating their reasoning for the sample size and identifying feasibility as the primary outcome. However, no justification was provided for why these specific progression criteria, i.e., the Green Limits, would render a future trial feasible and more importantly the distance between the Red and Green Limits was not justified. Given that we have no more information about the study we take the Green Limits as stated and assume there are good reasons to believe that $\ge 50\%$ recruitment, $\ge 70\%$ retention, etc. would lead to a future trial being feasible. But even so, the Red Limits are so far from the Green Limits that they can only provide limited information concerning whether a future trial will be feasible.

As an example, we focus on the recruitment and retention outcomes. Assume the pilot is successful, a full RCT is pursued and after a power analysis a sample size of *N* = 300 is needed with 100% retention. At the Green Limits, 70% retention and 50% recruitment then the study team will need to screen 860 participants to have 300 participants with complete data at the end of the study. Conversely at the Red Limits, the infeasible scenario, the study team would need to screen 3,000 participants to have *N* = 300 subjects at the end of the study.

If we take the hypothetical example further and assume three research coordinators who will screen participants and three years of active recruitment, each research coordinator would need to screen 1.84 participants per week on average to fully recruit at the Green limit combination. While at the Red Limit they would need to each screen 6.4 on average; more than tripling their work. To be clear, the combination that results in 6.4 per week is the boundary of the Red zone for both outcomes and therefore the study team would not want to proceed in this situation. However, that still implies that the amber zone contains many seemingly infeasible values. For instance, 4.3 participants per coordinator per week is firmly in the Amber zone (e.g., 50% retention, 30% recruitment) and yet this is still well over twice the work per research coordinator. This calls into question how reasonable the limits of the Amber and Red zone are. These limits, whether assessed according to the method by Avery et al. or used as the basis for a more formal method (e.g., Lewis, Wilson, or Montgomery) have direct implications on how well these methods can discriminate between feasible and infeasible future trials. In this example it is possible that the study team has considered these, or similar values, and will make ready use of the Amber zone to eliminate any combinations they could not handle, if so, that information should be included in the protocol so that readers and reviewers can better interpret and evaluate the results of the trial. If researchers do not carefully consider the impacts of the progression criteria they use, then they run the risk that proceeding to a future trial becomes a foregone conclusion.

### The analysis should justify the sample size

Sekome et al. [20] recently published the protocol for a feasibility study on lifestyle modifications for the control of hypertension. Feasibility was listed as one of several primary objectives, and the authors used a traffic light procedure. They proposed four progression criteria: recruitment, retention, engagement, and implementation. They provided traffic light rules for the first three criteria (Table 2) and will qualitatively assess the feasibility of implementation (Table 3).

**Table 3:** Progression criteria for Sekome et al

Outcome	Green limit	Red limit
Recruitment (rate)	$\ge 30$	$< 15$
Retention	$\ge 0.80$	$< 0.70$
Engagement	$\ge 0.80$	$<0.70$

The team plans to recruit 30 participants based on guidance from Browne through the Lancaster et al. citation. We can evaluate whether this sample will enable the study team to make a good decision about whether a future trial will be feasible. The set of feasible characteristics for recruitment, retention and engagement are $F = (30, 0.80, 0.80)$ and the infeasible set are $I = (15, 0.70, 0.70)$. Before proceeding, we note this plan suffers from the same problem raised in the previous section: the difference between the feasible and infeasible conditions, mainly regarding recruitment, is quite large. As a ‘‘go criteria’’ the team will proceed if they can recruit 30 or more per week and will definitely not proceed if they can recruit only 15 per week, practically the ‘‘no-go’’ condition would require twice as long to recruit the full sample.

Using the approach proposed by Montgomery et al. with a fixed sample size we can set a desired probability of proceeding given a future trial is feasible, denoted $P(Proceed \mid F)$, and for this example we let $P(Proceed \mid F) = 0.80$. This means that if the future trial is feasible, we want to have an 80% probability of proceeding. Then we can find the best, in this case smallest, probability we can obtain with this sample size of proceeding when a future trial is infeasible, denoted $P(Proceed \mid I)$. For this example, with *N* = 30 and the progression criteria in Table 2 we can achieve $P(Proceed \mid I) = 0.05$, that is with the decision rule the method provides there will only be a 5% chance of proceeding when all outcomes are at the Red limit. With the stated sample size and progression criteria the trial has excellent operating characteristics. The study team could have made a much stronger argument for why *N* = 30 was sufficient; instead of relying on published guidance, they can state the (impressive) rates at which they would make correct and incorrect decision.

For the sake of argument, we consider the effect of changing the questionable progression criteria for the recruitment rate. We keep *F* unchanged but let $I_{2} = (24, 0.70, 0.70)$. Practically, this would imply that recruitment at the $I_{2}$ rate would take 25% longer than at the *F* rate. We can still achieve $P(Proceed \mid F) = 0.80$ but we now have $P(Proceed \mid I_{2}) = 0.14$, a higher, although still acceptable, rate of proceeding when we should not. We can also see the impact of sample size by letting *N* = 50. We accordingly update the progression criteria to $F_{2} = (50, 0.8, 0.8)$ and $I_{3} = (40, 0.7, 0.7)$ so that again recruitment at the infeasible scenario will take 25% longer than at the feasible scenario. Increasing the sample size and keeping this potentially more meaningful distance between the recruitment rates would result in $P(Proceed \mid I_{3}) = 0.05$. Both the sample size and the progression criteria have large impacts on the operating characteristics that a feasibility study can achieve, and both should be carefully considered. Details of these analyses, including the Bayesian priors used are provided in the supplementary material.

## Some suggestions for improving feasibility study designs

Feasibility studies are critically important for assessing whether future trials are feasible. To make good decisions at the end of a feasibility study, the progression criteria, analysis plan, and sample size need to be carefully considered and tailored to the specific objectives of the trial.

Our suggestions for improving the design, and specifically the sample size justification, for feasibility studies closely mirror what is routinely done for RCTs. In an RCT, a typical approach is to first define the primary outcome and select a meaningful effect size and then to choose an analysis plan. Based on the effect size and analysis plan, we can identify the required sample size to reject the null hypothesis, with say 80% power while maintaining a type I error rate of 0.05.

For a feasibility study we should first specify the feasibility outcomes and meaningful progression criteria. (Note this probably requires knowing something about the design of the future trial, e.g., if you will need to recruit 500 participants in 5 years that has obvious bearing on your target recruitment rate). Second, choose an analysis plan that will address all progression criteria (or as needed different analysis plans for the constituent parts). And finally, select a sample size based on evaluating the progression criteria with the planned analysis to achieve some pre-specified operating characteristics across all outcomes. If one objective of the trial requires a larger sample size than the other objectives, then that sample size should be used for the full trial. In an RCT it is common to use power and type I error rate, in a feasibility study a similar pair of operating characteristics would be the probability of proceeding when a trial is feasible, and the probability of proceeding when it is not.

These simple suggestions would help remove much of the vagueness of using rules of thumb and guidance that may not be relevant for a particular study. It will require more upfront work on the part of study teams; however, it will also allow them to more compellingly argue why positive results can justify a future trial. Even in the common case where the sample size of a feasibility study is fixed beforehand this approach can be used to generate the best operating characteristics available for the sample size and will allow the study team and reviewers to make more informed decisions about feasibility.

## Conclusion

Feasibility studies are complex and often include preliminary estimates of efficacy and standard deviations, feasibility outcomes, and qualitative analyses. Any outcomes from these studies that will be used in some way to justify progression to, or planning of, a future definitive trial need to be incorporated in the determination of an appropriate sample size. If multiple aspects of the trial require different sample sizes (e.g., a sample size of 50 for recruitment and 30 for a qualitative endpoint) then the maximum sample size among the constituent parts should be chosen, otherwise, there can be little confidence that the study can successfully assess its objectives. In practice sample sizes for feasibility studies often have a hard cap based on funding, or time available for the study. Researchers can still determine the operating characteristics of their progression rules at the available sample size, and clearly state them, which will help readers and reviewers better interpret the results of the trial. Without justification published guidance can inspire little confidence that it covers the entirety of the study at hand. It is time for researchers to move beyond the simple use of published guidance for feasibility studies, and instead justify the sample size of their specific trial.

## Supplementary information

Supplementary Material 1.

## Data Availability

Not applicable.

## Abbreviation

RCT: Randomized controlled trial
