# Supplementary material for: Sample size justification in feasibility studies: moving beyond published guidance
Source: Pilot Feasibility Stud. 2025 Jun 23;11:88. doi: 10.1186/s40814-025-01675-9 (PMC12186404; doi:10.1186/s40814-025-01675-9)
Supplement: Supplementary file 1 — Supplementary Material 1. [file 40814_2025_1675_MOESM1_ESM.docx]

**Supplementary Material**

**Details of simulation example in section 2.1**

In the hypothetical example we examine whether using N = 24 as a sample size justification for estimating the standard deviation would also be sufficient to evaluate a target recruitment rate of 20 participants per month. We simulated data in R from a Poisson distribution with rate parameter 0.67 to simulate daily recruitment rates. Given that the sum of independent Poisson random variables is a Poisson random variable with the sum of the individual rate parameters (and making the simplifying assumption that a month is 30 days) then on average we would recruit 20 participants per month (30×0.67 = 20). We simulated daily recruitment 10,000 times to determine how many days it would take on average to recruit N = 24 participants with a recruitment rate of 20 per month. If for example, one of the simulations showed it took 38 days, we would used this to estimate the monthly recruitment rate as (24/38) × 30 = 18.94. We kept all 10,000 estimated recruitment rates and calculated the proportion that were more than 5 units away.

**Table S1**: Details on the pilot and feasibility studies used to generate Table 1

| **DOI** | **Sample Size** | **Justification for Sample** | **Number of Feasibility outcomes** | **Feasibility listed as primary** | **Feasibility as primary quote/justification** | **Notes** |
| --- | --- | --- | --- | --- | --- | --- |
| https://doi.org/10.1186/s40814-024-01447-x | 18 | Power for hypothesis test | 3 reported in Table 2 as "Key feasibility indicators for progression" | Yes | " … primary study objectives include the following: 1. To collect data to assess the feasibility of the intervention" |  |
| https://doi.org/10.1186/s40814-024-01452-0 | 30 | Guidance/Rule of thumb | 14 (8 are clearly quantitative) none listed as "Key". | Yes | Results section begins with subsection titled: "Primary outcomes (feasibility)" |  |
| https://doi.org/10.1186/s40814-024-01448-w | 120 | Previous studies | 3 (effect sizes and estimates of the standard deviation are not included) | Yes | " ...the primary aim of this study is to determine the feasibility of…" |  |
| https://doi.org/10.1186/s40814-023-01426-8 | 30 | Guidance/Rule of thumb | 1 clearly listed as primary (6 secondary) | Yes | "The primary outcome will be the proportion…" |  |
| https://doi.org/10.1186/s40814-023-01439-3 | 100 | Guidance/Rule of thumb | 4 (included as progression criteria) | Yes | "The primary outcome of this pilot trial is to determine its feasibility for a main trial." |  |
| https://doi.org/10.1186/s40814-024-01456-w | 30 | Guidance/Rule of thumb | 3 (4 included in Table 5 as progression criteria, 1 is qualitative) | Yes | "The primary objectives of the study are as follows: … c) to determine feasibility…" |  |
| https://doi.org/10.1186/s40814-023-01434-8 | 292 | Percentage of full trial | 5 ("minimum metrics to justify full trial") | Yes | "The primary aims for the pilot phase of the study are to assess recruitment feasibility, protocol adherence and data capture." | Internal pilot |
| https://doi.org/10.1186/s40814-023-01441-9 | 24 | Unclear (provide rationale for the N to recruit to achieve the target sample size, but not for the sample size itself.) | 6 (Listed in Table 2 with Traffic light approach) | Yes | See note. |  |
| https://doi.org/10.1186/s40814-023-01433-9 | 24 | Guidance/Rule of thumb | 4 (quantitative, many qualitative feasibility outcomes) | Yes | "The results will provide information on whether Get Back in its present format is feasible…" |  |
| https://doi.org/10.1186/s40814-023-01429-5 | 200 | Precision of confidence interval | 2 (see note) | Yes | "The primary outcome was the rate of recruitment to randomisation of participants to the trial..." | Recruitment rate is listed as primary but they authors state the study was powered based on retention, so both are counted here as the 'primary' feasibility outcomes. |
| https://doi.org/10.1186/s40814-023-01438-4 | 50 | Guidance/Rule of thumb | 7 (another qualitative) | Yes | "...the primary aim of the study is to assess the feasibility of conducting a study…" |  |
| https://doi.org/10.1186/s40814-024-01443-1 | 10 | None given | 10 (listed in Table 1) | Yes | No quote, but only feasibility is reported. |  |
| https://doi.org/10.1186/s40814-023-01437-5 | 80 | Guidance/Rule of thumb | 5 (and a qualitative focus group and efficacy measures) | Yes | "The primary aim of the current proposal is to assess the feasibility and acceptability…" |  |
| https://doi.org/10.1186/s40814-023-01425-9 | 40 | Guidance/Rule of thumb | 1 (several secondary outcomes) | Yes | "The primary outcome is the feasibility and acceptability…" |  |
| https://doi.org/10.1186/s40814-023-01435-7 | 14 | Pragmatic considerations | 4 (quantitative feasibility outcome, many other outcomes are listed) | Yes | "Primary: Feasibility [throughout trial]" although almost everything is listed as "primary". |  |
| https://doi.org/10.1186/s40814-024-01442-2 | 26 | Unclear (they think it will yield "meaningful" values) | 6 (listed as "Progression criteria") | Yes | Only reported outcomes are feasibility/acceptability |  |
| https://doi.org/10.1186/s40814-023-01428-6 | 50 | Unclear (none given) | 2 (several efficacy outcomes) | Yes | "The primary aim of the Koolungar Moorditj Healthy Skin pilot project is to inform the feasibility and design…" |  |
| https://doi.org/10.1186/s40814-023-01431-x | 91 | Power for hypothesis test | 3 | No |  |  |
| https://doi.org/10.1186/s40814-023-01430-y | 40 | None given | 3 (quantitative) | Yes |  | Study failed, only recruited 3 |
| https://doi.org/10.1186/s40814-023-01440-w | 48 | Hypothesis tests of feasibility endpoints | 5 (2 listed with hypothesis tests but 3 others will "inform the decision") | Yes | " … primary study objectives include the following: 1. To collect data to assess the feasibility of the intervention" |  |

Sample size: The sample size descriptions and detail differ greatly between studies. The values here are the target sample sizes for analysis, i.e., after any attrition. Some studies noted attrition, some did not for those that did not specifically mention attrition we treated the sample size as the target sample size.

**Details of Sekome Analysis**

For the analysis of the Sekome et al. protocol we used the method proposed by Montgomery et al, which we briefly review. Montgomery at al.’s method requires the research team to define sets of progression criteria denoted as F for feasible values and I for infeasible values, these are similar to the Red and Green limits used in the traffic light procedure proposed by Avery et al. Data is simulated with the specified sample size, N, and F values for the feasibility outcomes many times, e.g., 5,000. For each simulated data set the Bayesian posterior probability of all outcomes meeting their target rate is calculated. For example, in the Sekome analysis the target recruitment rate is 30. We can model this data using a Poisson distribution with a conjugate Gamma prior, specifically we let alpha = 150, and beta = 5, this gives a prior mean of 30 (mean is alpha over beta) with a ~50% prior probability of meeting the recruitment rate and a 5^th^ and 95^th^ percentile for the prior of 26 and 34 participants per week. Then with the simulated data for each of the 5,000 simulated recruitment rates we can calculate the Bayesian posterior probability of the rate being ≥ 30. And we can identify a cut point from these 5,000 Bayesian posterior probabilities such that if the true recruitment rate is 30, we would meet or exceed this probability 80% of the time. For this specific example the posterior probability of 0.017 would be met or exceeded 80% of the time. We can then generate data under condition I in the same way, with the same prior and calculate how many times out of 5,000 the posterior probability meets or exceeds 0.017. For this example, with N = 30, and I = (15, 0.7, 0.7) that cut point would only be met 5% of the time. (The cut point was calculated for the joint posterior of recruitment, retention and engagement. The priors for engagement and recruitment were Beta(1,1), i.e., Uniform.)

For the follow-up analyses we increased the sample size to N = 50, and accordingly updated the Gamma prior to have parameters alpha = 150, and beta = 3, this provides a mean of 50 and a 5^th^ and 95^th^ percentile of 43.5 and 56.9. We then used the same analysis to identify the cut point to give P(Proceed | F_2_) = 0.80 and determine how often it would be met under modified infeasible conditions I_2_ and I_3_.
